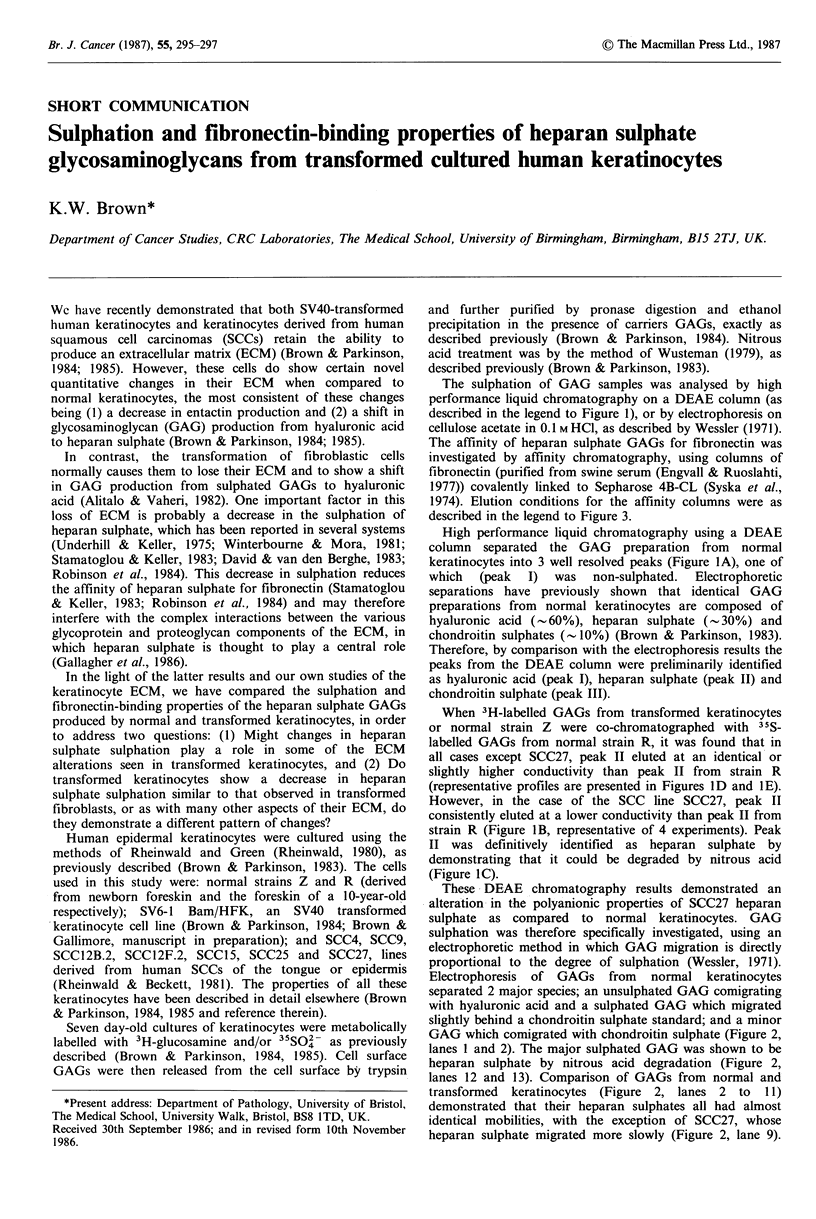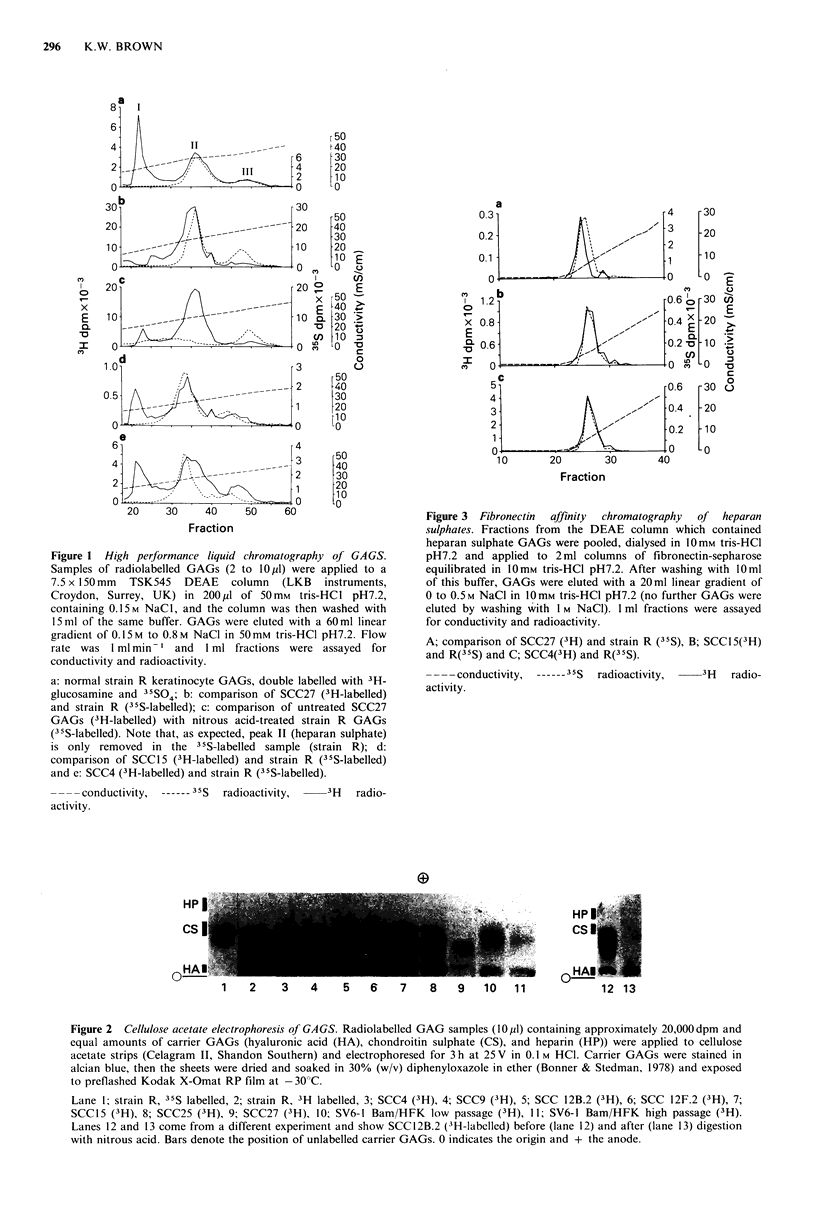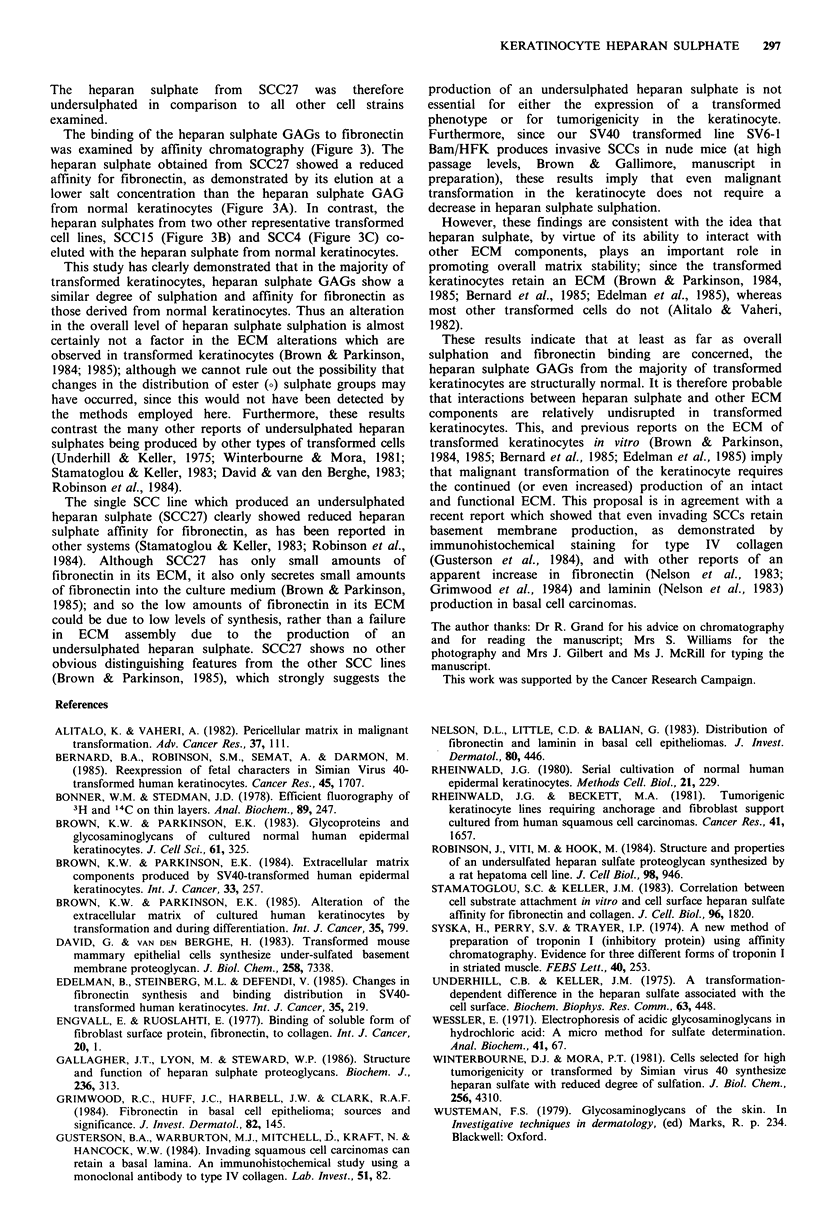# Sulphation and fibronectin-binding properties of heparan sulphate glycosaminoglycans from transformed cultured human keratinocytes.

**DOI:** 10.1038/bjc.1987.57

**Published:** 1987-03

**Authors:** K. W. Brown

## Abstract

**Images:**


Br. J. Cancer (1987), 55, 295–297 © The Macmillan Press Ltd., 1987

SHORT COMMUNICATION

### Sulphation and fibronectin-binding properties of heparan sulphate glycosaminoglycans from transformed cultured human keratinocytes

K.W. Brown*

*Department of Cancer Studies, CRC Laboratories, The Medical School, University of Birmingham, Birmingham, B15 2TJ, UK.*

We have recently demonstrated that both SV40-transformed human keratinocytes and keratinocytes derived from human squamous cell carcinomas (SCCs) retain the ability to produce an extracellular matrix (ECM) (Brown & Parkinson, 1984; 1985). However, these cells do show certain novel quantitative changes in their ECM when compared to normal keratinocytes, the most consistent of these changes being (1) a decrease in entactin production and (2) a shift in glycosaminoglycan (GAG) production from hyaluronic acid to heparan sulphate (Brown & Parkinson, 1984; 1985).

In contrast, the transformation of fibroblastic cells normally causes them to lose their ECM and to show a shift in GAG production from sulphated GAGs to hyaluronic acid (Alitalo & Vaheri, 1982). One important factor in this loss of ECM is probably a decrease in the sulphation of heparan sulphate, which has been reported in several systems (Underhill & Keller, 1975; Winterbourne & Mora, 1981; Stamatoglou & Keller, 1983; David & van den Berghe, 1983; Robinson *et al.*, 1984). This decrease in sulphation reduces the affinity of heparan sulphate for fibronectin (Stamatoglou & Keller, 1983; Robinson *et al.*, 1984) and may therefore interfere with the complex interactions between the various glycoprotein and proteoglycan components of the ECM, in which heparan sulphate is thought to play a central role (Gallagher *et al.*, 1986).

In the light of the latter results and our own studies of the keratinocyte ECM, we have compared the sulphation and fibronectin-binding properties of the heparan sulphate GAGs produced by normal and transformed keratinocytes, in order to address two questions: (1) Might changes in heparan sulphate sulphation play a role in some of the ECM alterations seen in transformed keratinocytes, and (2) Do transformed keratinocytes show a decrease in heparan sulphate sulphation similar to that observed in transformed fibroblasts, or as with many other aspects of their ECM, do they demonstrate a different pattern of changes?

Human epidermal keratinocytes were cultured using the methods of Rheinwald and Green (Rheinwald, 1980), as previously described (Brown & Parkinson, 1983). The cells used in this study were: normal strains Z and R (derived from newborn foreskin and the foreskin of a 10-year-old respectively); SV6-1 Bam/HFK, an SV40 transformed keratinocyte cell line (Brown & Parkinson, 1984; Brown & Gallimore, manuscript in preparation); and SCC4, SCC9, SCC12B.2, SCC12F.2, SCC15, SCC25 and SCC27, lines derived from human SCCs of the tongue or epidermis (Rheinwald & Beckett, 1981). The properties of all these keratinocytes have been described in detail elsewhere (Brown & Parkinson, 1984, 1985 and reference therein).

Seven day-old cultures of keratinocytes were metabolically labelled with $^{3}$H-glucosamine and/or $^{35}SO_4^{2-}$ as previously described (Brown & Parkinson, 1984, 1985). Cell surface GAGs were then released from the cell surface by trypsin and further purified by pronase digestion and ethanol precipitation in the presence of carriers GAGs, exactly as described previously (Brown & Parkinson, 1984). Nitrous acid treatment was by the method of Wusteman (1979), as described previously (Brown & Parkinson, 1983).

The sulphation of GAG samples was analysed by high performance liquid chromatography on a DEAE column (as described in the legend to Figure 1), or by electrophoresis on cellulose acetate in 0.1 M HCl, as described by Wessler (1971). The affinity of heparan sulphate GAGs for fibronectin was investigated by affinity chromatography, using columns of fibronectin (purified from swine serum (Engvall & Ruoslahti, 1977)) covalently linked to Sepharose 4B-CL (Syska *et al.*, 1974). Elution conditions for the affinity columns were as described in the legend to Figure 3.

High performance liquid chromatography using a DEAE column separated the GAG preparation from normal keratinocytes into 3 well resolved peaks (Figure 1A), one of which (peak I) was non-sulphated. Electrophoretic separations have previously shown that identical GAG preparations from normal keratinocytes are composed of hyaluronic acid (~60%), heparan sulphate (~30%) and chondroitin sulphates (~10%) (Brown & Parkinson, 1983). Therefore, by comparison with the electrophoresis results the peaks from the DEAE column were preliminarily identified as hyaluronic acid (peak I), heparan sulphate (peak II) and chondroitin sulphate (peak III).

When $^{3}$H-labelled GAGs from transformed keratinocytes or normal strain Z were co-chromatographed with $^{35}$S-labelled GAGs from normal strain R, it was found that in all cases except SCC27, peak II eluted at an identical or slightly higher conductivity than peak II from strain R (representative profiles are presented in Figures 1D and 1E). However, in the case of the SCC line SCC27, peak II consistently eluted at a lower conductivity than peak II from strain R (Figure 1B, representative of 4 experiments). Peak II was definitively identified as heparan sulphate by demonstrating that it could be degraded by nitrous acid (Figure 1C).

These DEAE chromatography results demonstrated an alteration in the polyanionic properties of SCC27 heparan sulphate as compared to normal keratinocytes. GAG sulphation was therefore specifically investigated, using an electrophoretic method in which GAG migration is directly proportional to the degree of sulphation (Wessler, 1971). Electrophoresis of GAGs from normal keratinocytes separated 2 major species; an unsulphated GAG comigrating with hyaluronic acid and a sulphated GAG which migrated slightly behind a chondroitin sulphate standard; and a minor GAG which comigrated with chondroitin sulphate (Figure 2, lanes 1 and 2). The major sulphated GAG was shown to be heparan sulphate by nitrous acid degradation (Figure 2, lanes 12 and 13). Comparison of GAGs from normal and transformed keratinocytes (Figure 2, lanes 2 to 11) demonstrated that their heparan sulphates all had almost identical mobilities, with the exception of SCC27, whose heparan sulphate migrated more slowly (Figure 2, lane 9).

*Present address: Department of Pathology, University of Bristol, The Medical School, University Walk, Bristol, BS8 1TD, UK.
Received 30th September 1986; and in revised form 10th November 1986.

**Figure 1** *High performance liquid chromatography of GAGS.* Samples of radiolabelled GAGs (2 to 10 μl) were applied to a 7.5×150 mm TSK545 DEAE column (LKB instruments, Croydon, Surrey, UK) in 200 μl of 50 mM tris-HCl pH7.2, containing 0.15 M NaCl, and the column was then washed with 15 ml of the same buffer. GAGs were eluted with a 60 ml linear gradient of 0.15 M to 0.8 M NaCl in 50 mM tris-HCl pH7.2. Flow rate was 1 ml min$^{-1}$ and 1 ml fractions were assayed for conductivity and radioactivity.

a: normal strain R keratinocyte GAGs, double labelled with $^3$H-glucosamine and $^{35}SO_4$; b: comparison of SCC27 ($^3$H-labelled) and strain R ($^{35}$S-labelled); c: comparison of untreated SCC27 GAGs ($^3$H-labelled) with nitrous acid-treated strain R GAGs ($^{35}$S-labelled). Note that, as expected, peak II (heparan sulphate) is only removed in the $^{35}$S-labelled sample (strain R); d: comparison of SCC15 ($^3$H-labelled) and strain R ($^{35}$S-labelled) and e: SCC4 ($^3$H-labelled) and strain R ($^{35}$S-labelled).

----conductivity, ------$^{35}$S radioactivity, ——$^3$H radioactivity.

**Figure 3** *Fibronectin affinity chromatography of heparan sulphates.* Fractions from the DEAE column which contained heparan sulphate GAGs were pooled, dialysed in 10 mM tris-HCl pH7.2 and applied to 2 ml columns of fibronectin-sepharose equilibrated in 10 mM tris-HCl pH7.2. After washing with 10 ml of this buffer, GAGs were eluted with a 20 ml linear gradient of 0 to 0.5 M NaCl in 10 mM tris-HCl pH7.2 (no further GAGs were eluted by washing with 1 M NaCl). 1 ml fractions were assayed for conductivity and radioactivity.

A; comparison of SCC27 ($^3$H) and strain R ($^{35}$S), B; SCC15($^3$H) and R($^{35}$S) and C; SCC4($^3$H) and R($^{35}$S).

----conductivity, ------$^{35}$S radioactivity, ——$^3$H radioactivity.

**Figure 2** *Cellulose acetate electrophoresis of GAGS.* Radiolabelled GAG samples (10 μl) containing approximately 20,000 dpm and equal amounts of carrier GAGs (hyaluronic acid (HA), chondroitin sulphate (CS), and heparin (HP)) were applied to cellulose acetate strips (Celagram II, Shandon Southern) and electrophoresed for 3 h at 25 V in 0.1 M HCl. Carrier GAGs were stained in alcian blue, then the sheets were dried and soaked in 30% (w/v) diphenyloxazole in ether (Bonner & Stedman, 1978) and exposed to preflashed Kodak X-Omat RP film at −30°C.

Lane 1; strain R, $^{35}$S labelled, 2; strain R, $^3$H labelled, 3; SCC4 ($^3$H), 4; SCC9 ($^3$H), 5; SCC 12B.2 ($^3$H), 6; SCC 12F.2 ($^3$H), 7; SCC15 ($^3$H), 8; SCC25 ($^3$H), 9; SCC27 ($^3$H), 10; SV6-1 Bam/HFK low passage ($^3$H), 11; SV6-1 Bam/HFK high passage ($^3$H). Lanes 12 and 13 come from a different experiment and show SCC12B.2 ($^3$H-labelled) before (lane 12) and after (lane 13) digestion with nitrous acid. Bars denote the position of unlabelled carrier GAGs. 0 indicates the origin and + the anode.

The heparan sulphate from SCC27 was therefore undersulphated in comparison to all other cell strains examined.

The binding of the heparan sulphate GAGs to fibronectin was examined by affinity chromatography (Figure 3). The heparan sulphate obtained from SCC27 showed a reduced affinity for fibronectin, as demonstrated by its elution at a lower salt concentration than the heparan sulphate GAG from normal keratinocytes (Figure 3A). In contrast, the heparan sulphates from two other representative transformed cell lines, SCC15 (Figure 3B) and SCC4 (Figure 3C) co-eluted with the heparan sulphate from normal keratinocytes.

This study has clearly demonstrated that in the majority of transformed keratinocytes, heparan sulphate GAGs show a similar degree of sulphation and affinity for fibronectin as those derived from normal keratinocytes. Thus an alteration in the overall level of heparan sulphate sulphation is almost certainly not a factor in the ECM alterations which are observed in transformed keratinocytes (Brown & Parkinson, 1984; 1985); although we cannot rule out the possibility that changes in the distribution of ester (o) sulphate groups may have occurred, since this would not have been detected by the methods employed here. Furthermore, these results contrast the many other reports of undersulphated heparan sulphates being produced by other types of transformed cells (Underhill & Keller, 1975; Winterbourne & Mora, 1981; Stamatoglou & Keller, 1983; David & van den Berghe, 1983; Robinson *et al.*, 1984).

The single SCC line which produced an undersulphated heparan sulphate (SCC27) clearly showed reduced heparan sulphate affinity for fibronectin, as has been reported in other systems (Stamatoglou & Keller, 1983; Robinson *et al.*, 1984). Although SCC27 has only small amounts of fibronectin in its ECM, it also only secretes small amounts of fibronectin into the culture medium (Brown & Parkinson, 1985); and so the low amounts of fibronectin in its ECM could be due to low levels of synthesis, rather than a failure in ECM assembly due to the production of an undersulphated heparan sulphate. SCC27 shows no other obvious distinguishing features from the other SCC lines (Brown & Parkinson, 1985), which strongly suggests the production of an undersulphated heparan sulphate is not essential for either the expression of a transformed phenotype or for tumorigenicity in the keratinocyte. Furthermore, since our SV40 transformed line SV6-1 Bam/HFK produces invasive SCCs in nude mice (at high passage levels, Brown & Gallimore, manuscript in preparation), these results imply that even malignant transformation in the keratinocyte does not require a decrease in heparan sulphate sulphation.

However, these findings are consistent with the idea that heparan sulphate, by virtue of its ability to interact with other ECM components, plays an important role in promoting overall matrix stability; since the transformed keratinocytes retain an ECM (Brown & Parkinson, 1984, 1985; Bernard *et al.*, 1985; Edelman *et al.*, 1985), whereas most other transformed cells do not (Alitalo & Vaheri, 1982).

These results indicate that at least as far as overall sulphation and fibronectin binding are concerned, the heparan sulphate GAGs from the majority of transformed keratinocytes are structurally normal. It is therefore probable that interactions between heparan sulphate and other ECM components are relatively undisrupted in transformed keratinocytes. This, and previous reports on the ECM of transformed keratinocytes *in vitro* (Brown & Parkinson, 1984, 1985; Bernard *et al.*, 1985; Edelman *et al.*, 1985) imply that malignant transformation of the keratinocyte requires the continued (or even increased) production of an intact and functional ECM. This proposal is in agreement with a recent report which showed that even invading SCCs retain basement membrane production, as demonstrated by immunohistochemical staining for type IV collagen (Gusterson *et al.*, 1984), and with other reports of an apparent increase in fibronectin (Nelson *et al.*, 1983; Grimwood *et al.*, 1984) and laminin (Nelson *et al.*, 1983) production in basal cell carcinomas.

The author thanks: Dr R. Grand for his advice on chromatography and for reading the manuscript; Mrs S. Williams for the photography and Mrs J. Gilbert and Ms J. McRill for typing the manuscript.

This work was supported by the Cancer Research Campaign.

### References

ALITALO, K. & VAHERI, A. (1982). Pericellular matrix in malignant transformation. *Adv. Cancer Res.*, **37**, 111.

BERNARD, B.A., ROBINSON, S.M., SEMAT, A. & DARMON, M. (1985). Reexpression of fetal characters in Simian Virus 40-transformed human keratinocytes. *Cancer Res.*, **45**, 1707.

BONNER, W.M. & STEDMAN, J.D. (1978). Efficient fluorography of $^{3}H$ and $^{14}C$ on thin layers. *Anal. Biochem.*, **89**, 247.

BROWN, K.W. & PARKINSON, E.K. (1983). Glycoproteins and glycosaminoglycans of cultured normal human epidermal keratinocytes. *J. Cell Sci.*, **61**, 325.

BROWN, K.W. & PARKINSON, E.K. (1984). Extracellular matrix components produced by SV40-transformed human epidermal keratinocytes. *Int. J. Cancer*, **33**, 257.

BROWN, K.W. & PARKINSON, E.K. (1985). Alteration of the extracellular matrix of cultured human keratinocytes by transformation and during differentiation. *Int. J. Cancer*, **35**, 799.

DAVID, G. & VAN DEN BERGHE, H. (1983). Transformed mouse mammary epithelial cells synthesize under-sulfated basement membrane proteoglycan. *J. Biol. Chem.*, **258**, 7338.

EDELMAN, B., STEINBERG, M.L. & DEFENDI, V. (1985). Changes in fibronectin synthesis and binding distribution in SV40-transformed human keratinocytes. *Int. J. Cancer*, **35**, 219.

ENGVALL, E. & RUOSLAHTI, E. (1977). Binding of soluble form of fibroblast surface protein, fibronectin, to collagen. *Int. J. Cancer*, **20**, 1.

GALLAGHER, J.T., LYON, M. & STEWARD, W.P. (1986). Structure and function of heparan sulphate proteoglycans. *Biochem. J.*, **236**, 313.

GRIMWOOD, R.C., HUFF, J.C., HARBELL, J.W. & CLARK, R.A.F. (1984). Fibronectin in basal cell epithelioma; sources and significance. *J. Invest. Dermatol.*, **82**, 145.

GUSTERSON, B.A., WARBURTON, M.J., MITCHELL, D., KRAFT, N. & HANCOCK, W.W. (1984). Invading squamous cell carcinomas can retain a basal lamina. An immunohistochemical study using a monoclonal antibody to type IV collagen. *Lab. Invest.*, **51**, 82.

NELSON, D.L., LITTLE, C.D. & BALIAN, G. (1983). Distribution of fibronectin and laminin in basal cell epitheliomas. *J. Invest. Dermatol.*, **80**, 446.

RHEINWALD, J.G. (1980). Serial cultivation of normal human epidermal keratinocytes. *Methods Cell. Biol.*, **21**, 229.

RHEINWALD, J.G. & BECKETT, M.A. (1981). Tumorigenic keratinocyte lines requiring anchorage and fibroblast support cultured from human squamous cell carcinomas. *Cancer Res.*, **41**, 1657.

ROBINSON, J., VITI, M. & HOOK, M. (1984). Structure and properties of an undersulfated heparan sulfate proteoglycan synthesized by a rat hepatoma cell line. *J. Cell Biol.*, **98**, 946.

STAMATOGLOU, S.C. & KELLER, J.M. (1983). Correlation between cell substrate attachment *in vitro* and cell surface heparan sulfate affinity for fibronectin and collagen. *J. Cell. Biol.*, **96**, 1820.

SYSKA, H., PERRY, S.V. & TRAYER, I.P. (1974). A new method of preparation of troponin I (inhibitory protein) using affinity chromatography. Evidence for three different forms of troponin I in striated muscle. *FEBS Lett.*, **40**, 253.

UNDERHILL, C.B. & KELLER, J.M. (1975). A transformation-dependent difference in the heparan sulfate associated with the cell surface. *Biochem. Biophys. Res. Comm.*, **63**, 448.

WESSLER, E. (1971). Electrophoresis of acidic glycosaminoglycans in hydrochloric acid: A micro method for sulfate determination. *Anal. Biochem.*, **41**, 67.

WINTERBOURNE, D.J. & MORA, P.T. (1981). Cells selected for high tumorigenicity or transformed by Simian virus 40 synthesize heparan sulfate with reduced degree of sulfation. *J. Biol. Chem.*, **256**, 4310.

WUSTEMAN, F.S. (1979). Glycosaminoglycans of the skin. In *Investigative techniques in dermatology*, (ed) Marks, R. p. 234. Blackwell: Oxford.